# Acute management of vascular air embolism

**DOI:** 10.4103/0974-2700.55330

**Published:** 2009

**Authors:** Nissar Shaikh, Firdous Ummunisa

**Affiliations:** Department of Anesthesia/ICU, Hamad Medical Corporation, Doha, Qatar

**Keywords:** Vascular air embolism, high risk surgeries, pressure gradient, decompression syndrome, transesopageal echocardiogram, hyperbaric oxygen, dysbarism

## Abstract

Vascular air embolism (VAE) is known since early nineteenth century. It is the entrainment of air or gas from operative field or other communications into the venous or arterial vasculature. Exact incidence of VAE is difficult to estimate. High risk surgeries for VAE are sitting position and posterior fossa neurosurgeries, cesarean section, laparoscopic, orthopedic, surgeries invasive procedures, pulmonary overpressure syndrome, and decompression syndrome. Risk factors for VAE are operative site 5 cm above the heart, creation of pressure gradient which will facilitate entry of air into the circulation, orogenital sex during pregnancy, rapid ascent in scuba (self contained underwater breathing apparatus) divers and barotrauma or chest trauma. Large bolus of air can lead to right ventricular air lock and immediate fatality. In up to 35% patient, the foramen ovale is patent which can cause paradoxical arterial air embolism. VAE affects cardiovascular, pulmonary and central nervous system. High index of clinical suspicion is must to diagnose VAE. The transesophgeal echocardiography is the most sensitive device which will detect smallest amount of air in the circulation. Treatment of VAE is to prevent further entrainment of air, reduce the volume of air entrained and haemodynamic support. Mortality of VAE ranges from 48 to 80%. VAE can be prevented significantly by proper positioning during surgery, optimal hydration, avoiding use of nitrous oxide, meticulous care during insertion, removal of central venous catheter, proper guidance, and training of scuba divers.

## INTRODUCTION

Vascular air embolism (VAE) is known since early 19^th^ Century but the interest and reporting of VAE significantly increased in last three decade. Most of the episodes of VAE are preventable if meticulous precaution taken or at least detected early and managed properly. It is important that all acute care physicians should be aware of this medical emergency. VAE can be venous or arterial, both condition can be differentiated by mechanism of air entry as well as site of embolization. The venous or pulmonary air embolism is air entry in systemic venous circulation reaching the right ventricle while arterial air embolism occurs due to entry of air in to the arterial circulation and potentially life threatening as it can lead to circulatory deficiency in the body organ with poor collateral circulation. Here we will review the VAE in the following sub-headings.

## DEFINITION

VAE is the entrainment of air (or exogenously delivered gas) from open operative field or communication with environment into the venous or arterial vasculature, producing systemic effects.[1] Or production of air bubbles in circulation due to dysbaric barotrauma in scuba (self contained underwater breathing apparatus) divers, astronauts and aviators, air enters venous or arterial circulation causing systemic air embolism.

## EPIDEMIOLOGY

Exact incidence of VAE is difficult to know as the sub clinical cases will be unnoticed. VAE probably most common embolic event to occur during the intra-operative period. In neurosurgical patients the incidence of VAE varies from 10 to 80%.[2] While in obstetric- gynaecological surgeries VAE can occur from 11 to 97% of the patients.[3] In laproscopic surgical patients it is reported to occur in up to 69% of the patients.[4] In orthopedic surgeries the incidence of VAE can be 57%.[5] During invasive monitoring catheter insertion the incidence of VAE are less than 2%.[6] Approximately, 7% of penetrating chest trauma patient will have VAE.[7] Few case reports of VAE after barotraumas[8] and use of pressure infuser bag.[9] In scuba (self contained underwater breathing apparatus) divers air embolism is second common fatal cause, the incidence are 7/100,000 dives.[10]

## ETIOLOGY

As mentioned VAE is probably most common embolic event to occur during surgical procedure.[11] First and primary etiology of VAE is surgical procedure where the operative site is above the level of heart, such as sitting neurosurgical procedure, posterior fossa surgery,[12] obstetric procedure,[13] and orthopedic surgeries. [14] Second etiological reason is iatrogenic creation of pressure gradient which facilitate air entry into the circulation such as during insertion of central venous catheter.[15] Third etiological cause is mechanical insufflations or pressure infusion such as laparoscopic surgeries[16] and gastrointestinal endoscopies. [17] Fourth reason for VAE is scuba diving, aviators, astronauts (due to dysbarism or changes in the ambient barometric pressures) and positive pressure ventilation.[18] The other etiological factors are blunt and penetrating trauma to chest[19] and head.[20]

## RISK FACTORS

In most of VAE episodes there are clear risk factors.

1. When operative site is more than 5 cm above right atrium.
2. Numerous larger, noncompressed venous channels in the surgical field.
3. Creation of pressure gradient for air entry into the circulation. A pressure difference of 5 cm of H_2_O across a 14 gauge needle can allow 100 ml of air/sec to enter into the circulation.[21]
4. Orogenital sex during pregnancy and puerperium, as there will be pelvic venous congestion and larger capacity of vagina during pregnancy, there is great risk for VAE.[22]
5. Barotrauma or trauma to chest causes alveolar rupture [Figure 1] into small vein and capillaries leading to direct air entry into the circulation.[23]

**Figure 1:**
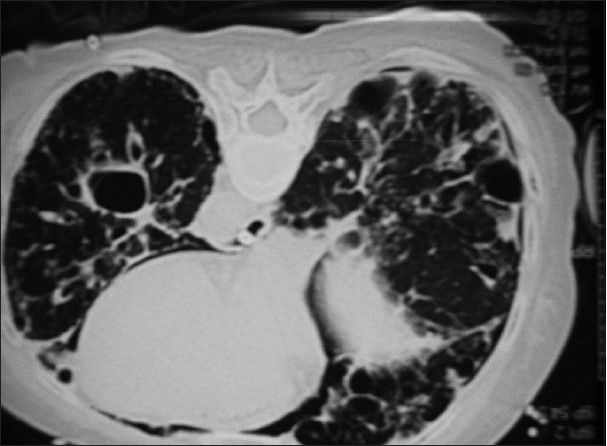
Baro-trauma leading to pneumothorax and free air
6. During insertion and removal of central venous catheter the factors increasing the risk of VAE such as fracture or detachment of catheter, failure to occlude the needle hub, deep inspiration, hypovolumia, and up-right position.
7. Risk factors which increases the incidence of air embolism in scuba divers are, too rapid ascent rate, diving for too long, diving at great depth, hard exercise at depth, dehydration, old age, obesity, alcohol, and cold water.[24]
8. Risk factors for air embolism in aviator are bronchogenic cyst or cystic air filled lesion in the lung.

## PATHOPHYSIOLOGY

Smaller amount of air in the circulation does not cause any clinical manifestations as it is broken and get absorbed from circulation.

Moderate amount of air causes pulmonary vascular injury leading to pulmonary hypertension and permeability pulmonary edema.[25]

Large bolus of air in venous system can cause an air lock in right side of the heart leading to right ventricular flow obstruction and death.[26]

**Figure 2:**
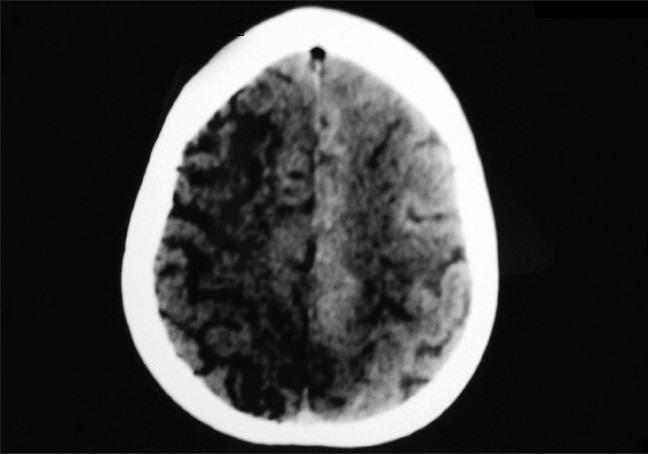
Air in brain due to paradoxical air embolism

**Figure 3:**
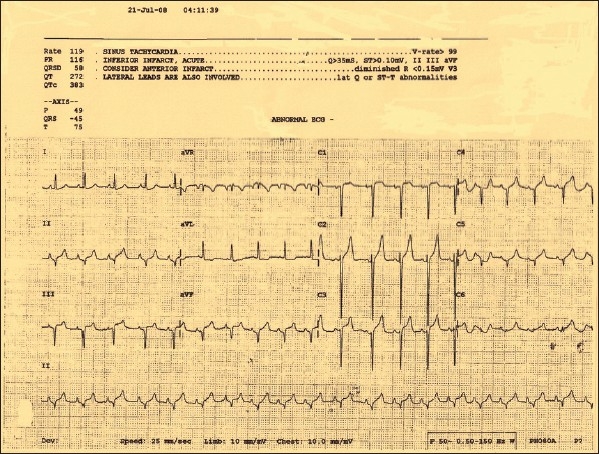
ECG changes in a patient with paradoxical air embolis

In up to 35% of patient due to the presence of patent foramen ovale, the air passes from right side to the left side of the heart and leading to systemic air emboliztion this is termed as paradoxical embolism.[27] In such cases cerebral [Figure 2] and myocardial ischaemia [Figure 3] can result from emboliztion of cerebral or coronary circulation.[28]

The fatal amount of air in human is reported to be either 200 and 300 ml bolus or 3-5 ml/kg.[29]

The pathophysiology of decompression syndrome in scuba divers is different from other iatrogenic air embolism. These dysbaric changes affect more in divers than high altitude climbers or astronauts as the density of water is 100 times more at the sea level and the ambient pressure is linear to the depth of submergence; to understand the pulmonary overpressure syndrome and decompression syndrome pathology we should know the law of physics concern with the partial pressure of gases in relation to the ambient pressure and gas solubility. The ambient pressure changes or dysbarism causes remarkable changes in the physiology of the breathing gases; (1) according to Dalton's law, the partial pressure of the divers breathing gases increases proportionally to the increase in ambient pressure as the depth increases; (2) these exposure to the supernormal pressures of breathing gases will lead to proportionally supernormal amount of these gases dissolved into the divers body tissues and this may lead to nitrogen narcosis or oxygen toxicity.[30]

The third type of changes in the body of the divers occurs which can be explained by the Boyle's law; that is the amount of gas compressed in the body cavities inversely related to the increased ambient pressure. These gases will expand due to ascend and decrease in ambient pressure leading to the barotrauma in non-distensible body tissues (in middle ear and sinuses) and distensible body tissues (bowel and lung) leading to pulmonary overpressurization and barotrauma which ultimately causes rupture of alveoli, pneumothorax, pneumomediastinum, arterial air embolism and decompression syndrome.[31]

The gas bubbles at the capillary level causes direct endothelial injury leading to post capillary constriction, platelets aggregation around the air bubbles with neutrophilic sequestration and inflammatory changes.

## CLINICAL MANIFESTATIONS

Presentation of VAE varies according to the nature, volume and speed of air entrainment into the circulation. The main affected systems in VAE are cardiovascular, respiratory, and central nervous system.

The cardiac manifestations are chest pain, bradyarrhythmias or tachyarrhythmias, increased filling pressure due to right sided heart failure and in late stage typical mill-wheel murmur. Electrocardiogram may show s-t segment changes or right ventricular strain pattern.[32]

The pulmonary changes include dyspnoea, tachypnoea and ‘gasp’ reflex as a result of acute hypoxaemia or even pulmonary edema. Patient may have reduced lung compliance, increased dead space and acute shunting leading to hypoxaemia and hypercarbia.[33]

Neurological manifestations are due to two reasons; first the cardiovascular collapse causing reduced cardiac output leading to cerebral hypo-perfusion, secondly direct paradoxical cerebral embolism[26] may occur through patent foramen ovale.

In divers there will be two types of injuries. First one is due to changes that leads to expansion of the breathing gases causing pulmonary over inflation syndrome; second type of injuries occur due to changes in ambient pressure leading to bubble formation initially in the body tissue then entering in to the circulation causing to decompression syndrome (DCS) or decompression sickness.

The pulmonary over inflation syndrome ranges from minor parenchymal lung injury causing local bleed, pneumothorax and less commonly but potentially dangerous, over expansion of alveoli leading to rapture and entry of air into the pulmonary venoules and arterioles causing systemic air embolism, clinically these can present as cerebrovascular accidents, paralysis, convulsion, coma, and may be associated with cardiovascular instability.[31]

DCS is the clinical manifestation associated with liberation of gas originally held in the solution into a free gas phase within the tissues as a result of decrease in the barometric pressures; these bubbles finally reaches venules.

**Figure 4:**
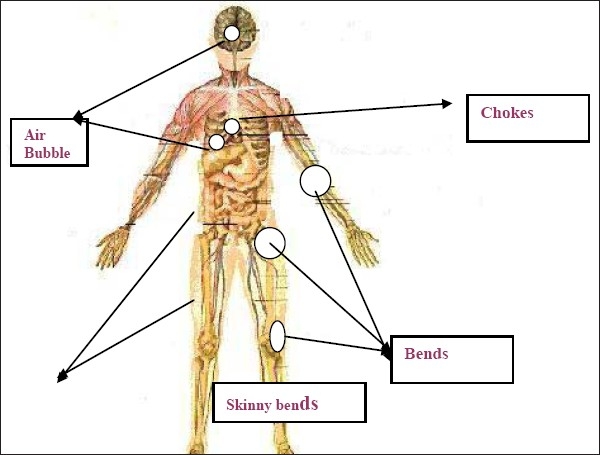
Main clinical manifestation of decompression syndrome

The DCS manifest as bends chokes and skinny bends [Figure 4]. The bends is boring pain in major joints (hip, elbow, and knee) of the divers mostly due to increase in intramedullary pressure at the ends of long bones and gas phase separation along ligaments and the tendon sheath causing sever pain. Chokes is substernal burning, cough and shortness of breath with or without haemodynamic instability. The main explanation for chokes is that the extreme high load of venous gas emboli in the pulmonary artery leading to increased in pressure on right side of the heart. Shinny bends are the coutaneous manifestation of DCS. The dangerous form of DCS is affection of spinal cord leading to ascending paraesthesia and associated bowel and urinary bladder dysfunction.[34]

## DETECTION OF VASCULAR AIR EMBOLISM

High index of suspicion in high risk cases is the cornerstone for diagnosis of VAE.

VAE should be considered when unexplained hypotension or sudden decrease in end-tidal CO_2_ level occurs intraoperatively in high risk cases. If patient complains of short of breath during or immediately after insertion or removal of central venous catheter. In cesarean section patients who suddenly develops hypotension and hypoxia after delivery of the fetus.

Various monitoring devices can help in early detection of air in the vascular system.

The transesophageal echocardiography is the most sensitive monitoring; it can detect 0.02 ml/kg of air injected by bolus administration or air bubbles as small as 5-10 microns.[35]

Precordial doppler ultrasound is the most sensitive non-invasive monitoring which can detect as little as 0.05 ml/kg of air.[36]

End tidal nitrogen (ETN2) can show the changes 30-90 seconds earlier than end-tidal carbon dioxide (ETCO2) changes.[37]

End-tidal carbon dioxide (ETCO2) is the most common and easily available monitoring, which will reveal sudden decrease in level in event of VAE.[38]

Transcranial doppler[39] and ECG (Electrocardiogram) changes are also used to guide the diagnosis of VAE. Pulmonary artery catheter will show rise in pulmonary artery pressure, its sensitivity is only 15%. Ventilation–perfusion scan findings are similar to those found in pulmonary thromboembolism, however, air embolism causes perfusion defect which get resolved rapidly usually within 24 hours. Computed Tomography (CT) chest will show air in central venous system, ventricles, pulmonary artery or pneumothorax [Figure 1]. CT brain may show intravascular air with or without infarction [Figure 2].[40]

## DIFFERENTIAL DIAGNOSIS

VAE should be differentiated from acute coronary syndrome, cardiogenic shock, cerebrovascular accidents, and pulmonary embolism.

## TREATMENT

The goal of treatment in VAE is to prevent further air entry, reduction in volume of air entrained, and haemodynamic support. Immediately covering the surgical field with saline soaked dressing and if possible tilting the Table may help in preventing further air entrainment. In sitting position surgeries, by jugular venous compression, it is documented that it limits or restrict the air entry into chest circulation.[41]

Administration of 100% oxygen will maximize the patient's oxygenation as well as reduces embolus volume by eliminating nitrogen.[42]

By maintaining the systemic arterial pressure with optimal fluid status and inotropic support to the heart will help by keeping the patient stable[43] Air lock in right side of heart may be relieved by partial left lateral decubitus position.[44]

Trendelenburg's position as a favorable placement to optimize haemodynamics is now controversial.[45]

Aspiration of air from right atrium is possibly best treatment to improve the haemodynamic parameters immediately and this can be done, with use of Bunegin-Albin multiorifice catheter up to 60% success rate.[46]

Rapid cardiopulmonary resuscitation with chest compression demonstrated to be effective in massive VAE which result in cardiac standstill.[47]

Hyperbaric oxygen therapy in the cases of VAE is beneficial as it causes compression of existing air bubbles, by establishing a high diffusion gradient to speed dissolution of bubbles and by improving oxygenation in the ischemic tissues.[48] In hyperbaric therapy, patient inspires 100% oxygen at pressure above that of atmosphere at sea level with achieving an arterial partial pressure of oxygen greater than 2000 mm of Hg. In decompression syndrome, patients should be transported in supine position to minimize risk of cerebral embolization. The early compression therapy has better results, as per Boyle's law, the compression by hyperbaric oxygen therapy will decrease the bubble size. Most commonly initial schedule of recompression is for 5-hours with intermittent air break. If sign and symptoms not relieved, therapy can be repeated once or twice daily and most of the patients required one to three therapeutic sessions.[49]

Anticoagulation therapy with heparin in patients with air embolism decreases the severity of the disease, if treated with heparin before air embolization.[50] As steroids do not have any effect on cytotoxic brain edema which occurs in the patients with air emboilsm, the use of steroid is controversial.[51] Interestingly, prophylactic lidocain is effective in reducing the gas embolism effect on brain, it deceases brain edema in experimental animals. Various case reports suggesting that the lignocain has beneficial effect in patient with decompression syndrome.[52]

## MORBIDITY AND MORTALITY

Morbidity and mortality in VAE is directly related to volume of air entrainment, rate of accumulation of air, and position of the patient at the time of VAE. Mortality of VAE ranges from 48 to 80%.[53]

## PREVENTION

Instead of sitting position for surgeries the alternative ‘park bench’ position provides adequate surgical conditions and to take extra-precautions and meticulous monitoring in patients with documented right to left shunt.[54] During caesarean section, 15 degree left tilt position facilitates entry of air into the circulation; hence institution of 50 degree reverse trendelenburg position decreases the chances of VAE from 44 to 1%.[55] During insertion and removal of central venous catheter one has to take care of all these risk factors for VAE to prevent its occurrences.[56] Optimizing the volume status will prevent the wide gradient between right atrium and entrain vein and hence decreasing the risk of VAE.[57] Avoidance of use of nitrous oxide will help in preventing VAE.[42] The use of positive end expiratory pressure (PEEP) in preventing VAE is controversial.[58]

The use of dive computers, published Tables and personal software algorithm recommended limiting the dive duration, at given depth and prescribing maximum ascent and decompression stops for various combinations of depth, dive time, and breathing gases which will be helpful in preventing decompression sickness in divers.

## CONCLUSION

Vascular air embolism (VAE) is preventable critical medical emergency. Apart from sitting position neurosurgical procedures, VAE is common in obstetric and laproscopic surgeries. It is the most feared complication in scuba divers. Small amount of air in the circulation get absorbed but large bolus of air can cause air lock in the heart causing sudden death. Clinical manifestation of VAE is mainly due to the involvement of respiratory, cardiovascular, and central nervous system. In scuba divers, the barometric pressure changes will lead to change in breathing gas solubility and expansion causing bubble formation in body tissue and circulation.

Paradoxical arterial air embolism occurs through patent foramen ovale causing significant end-organ damage. Precordial doppler ultrasound is the most sensitive method in detection of air embolism, but high index of suspicion in the high risk patients and knowledge about VAE are the corner stone for diagnosis of vascular air emboilsm. The goal of treatment of VAE is to prevent further air entry in the circulation, reduction in volume of air entrained and haemodynamic support. Aspiration of air from heart will immediately improve the haemodynaic parameters, but use of Trendelenburg position is controversial. The early use of hyperbaric oxygen therapy is vital in the treatment of VAE. To prevent occurrence of VAE the proper positioning during surgery, optimal hydration, and meticulous precautions during insertion and removal of central venous catheter is of vital importance. Use of diving computers, proper training and knowledge will prevent the decompression syndrome in the scuba divers.
